# Longevity regulation in flies: A role for p53

**DOI:** 10.18632/aging.100010

**Published:** 2009-01-10

**Authors:** Lawrence A. Donehower

**Affiliations:** Department of Molecular Virology and Microbiology, Department of Molecular and Cellular Biology, Baylor College of Medicine, Houston, TX 77030, USA

The p53 gene is justly famous for its role as a tumor suppressor. Loss of its function through structural alterations (inactivating mutations, deletions) occurs in roughly half of all human cancers, making it the most frequently mutated cancer-associated gene [1]. Yet p53 does not function only to suppress cancer, as p53 homologues are present in short-lived invertebrates such as worms (*Caenorhabditis elegans*) and flies (*Drosophila melanogaster*) that do not develop cancer [2]. p53 is known as the "guardian of the genome" and plays an important role in maintaining genomic integrity in response to cellular damage and stress [3]. In response to DNA damage, p53 is activated and can induce cell cycle arrest of actively dividing cells, allowing time for repair of damage before re-entry into the cell cycle. In other cases, where damage is extensive, p53 can induce apoptosis, preventing the propagation of damaged or dysfunctional cells. It is likely that p53 protects flies and worms primarily by regulating apoptosis during embryogenesis. Yet p53 may play another role in flies as a longevity regulator, as illustrated by a paper by Helfand and colleagues in this issue [4].

How does the capacity of p53 to regulate genomic integrity relate to its potential ability to regulate aging and longevity? The first hints were obtained from genetically engineered mouse models that exhibited accelerated aging phenotypes and shortened longevity [5,6]. Such models frequently displayed evidence of an elevated p53 response, suggesting that enhanced p53 activity might be partially responsible for these premature aging phenotypes. More direct evidence for p53 involvement in longevity came from studies on two mouse models that expressed truncated mutant forms of p53 [7,8]. These mouse mutants, one generated by my laboratory, were resistant to cancer, yet had significantlyshortened longevity accompanied by a number of premature aging phenotypes. We hypothesized that the truncated versions of p53 were causing aberrant regulation of resident wildtype p53, resulting in an augmented p53-mediated anti-proliferative response [9]. This enhanced p53 response may inhibit functionality of stem and progenitor cell compartments, leading to some of the tissue atrophies and dysfunctions that accompanied the premature aging and shortened longevity phenotypes. A number of laboratories have shown that increased activity of tumor suppressors such as p53 and p16^INK4a^ in stem cell compartments can lead to reduced stem cell self renewal, tissue reconstitution function, and early tissue atrophies [10-14]. It should be noted that transgenic mice with one or two copies of normally regulated p53, while showing cancer resistance, did not have altered longevity [15]. However, if an additional copy of the intact tumor suppressor p19^ARF^ (which activates p53) was co-expressed along with an additional copy of p53, aging could actually be delayed [16]. Thus, normally regulated, though augmented p53 function may be longevity enhancing, while aberrantly regulated, but enhanced p53 activity is detrimental to longevity (though both conditions inhibit cancer formation).

The clear influence of p53 on mouse longevity, either lengthening or shortening it, led Helfand and colleagues to investigate the role of the *Drosophila* p53 homologue, Dmp53, in longevity [17]. They found that null Dmp53 flies, while viable, are sickly and have a reduced lifespan, probably due to early negative effects on embryonic development. However, when two dominant negative mutants of p53 (shown to significantly inhibit wildtype p53 transactivation activity) were expressed in the neuronal cells of Drosophila, longevity was increased by up to 58%. Even if the dominant negative (DN) p53 was expressed only in adult neurons, longevity extension of 26% could be achieved. That this longevity effect was tissue-specific was shown by the fact that DN Dmp53 expression in muscle or fat body cells resulted in no longevity enhancement. Thus, DN dmp53 mediated reduction of p53 activity in specific tissues could lead to delayed aging and extended longevity.

Insights into the mechanisms of this neuronal Dmp53 effect were provided by the finding that calorie restriction (CR) of the neuronal DN Dmp53 flies does not provide any additional lifespan extension beyond that observed in non-restricted DN Dmp53 flies [17]. Calorie restriction is a consistent extender of longevity in worms, flies and mice. The non-additive effects of DN p53 and CR argue that p53 signaling and calorie restriction are operating in the same pathway or affecting the same pathway. In worms and flies, the pathway most consistently associated with longevity effects has been the insulin signaling pathway [18]. *C. elegans* and *Drosophila* mutations in insulin signaling pathway members that result in reduced insulin signaling often increase longevity and enhance stress resistance, probably in part through enhanced dFoxO activity [19]. The effects of calorie restriction phenocopy those observed in insulin signaling mutants, and in flies the affected pathways may overlap. An important advance by the Helfand laboratory was the recent demonstration that specific expression of the DN Dmp53 transgene in the 14 neurons of the brain that produce insulin-like peptides extends lifespan to a similar extent as pan-neuronal expression [20]. The primary effect of DN Dmp53 in the insulin-producing neurons was on reducing production of insulin-like peptide 2 (dILP2), while other dILPs remained unaffected. It was demonstrated that reduction of dILP2 production was sufficient to inhibit downstream insulin signaling, as evidenced by reduced PI-3 kinase function in fat bodies of both larval and adult flies. Moreover, increased dFoxO nuclear accumulation was observed in fat body cells, a key downstream readout for attenuated insulin signaling, as increased nuclear FoxO is associated with increased stress resistance [19]. Thus, alterations in p53 signaling in a specific subset of secretory neurons were shown to cause major effects on insulin signaling pathways in critical target organs such as the fat body.

The most recent extension of the Helfand laboratory findings are described in this issue [4]. Here, they used an inducible GeneSwitch System to temporally manipulate expression of the DN Dmp53 transgene. Turning on expression of the DN Dmp53 construct at 10 and 20 days of age in adult Drosophila brains led to 29% and 12% longer life spans, respectively, compared to control flies. While less than the 47% longevity extension observed in flies with constitutive lifelong DN Dmp53 expression, it demonstrates that the transgene longevity extension is not solely due to positive embryonic or early life effects. The reverse experiment of turning off DN Dmp53 expression also reduced lifespan extension proportionate to the age of turnoff.

The earlier interactions of calorie restriction and DN Dmp53 longevity effects were further examined by testing interactions of DN Dmp53 and dSir2 overexpression. Overexpression of the histone deace-tylase Sir2 has been associated with increased lifespan in yeast, worms, and flies [21]. Moreover, it is generally believed that CR acts in part through Sir2 activation, since Sir2 overexpression and CR longevity extension effects are not additive in flies [22]. Here, the authors showed that Sir2 overexpressing DN Dmp53 flies showed the same longevity as Sir2 overexpressing flies, indicating no additive effects of the two alleles and that they were likely interacting in the same pathway relating to CR. This result was confirmed by treament of control flies and DN Dmp53 flies with resveratrol, a molecular activator of Sir2 that has been shown to extend lifespan in yeast, worms, and flies. No significant difference in fly lifespan extension was observed among DN Dmp53 flies, resveratrol treated control flies, and resveratrol treated DN Dmp53 flies, suggesting that CR, dSir2 and DN Dmp53 act through similar pathways of longevity extension.

The pathway interactions of dSir2 and Dmp53 were confirmed at the biochemical level by showing through co-immunoprecipitation experiments in fly head lysates that Dmp53 and Sir2 physically interact with each other. In addition, purified recombinant dSir2 protein deacetylated acetylated peptides corresponding to known sites of Dmp53 acetylation in an NAD-dependent manner. Co-transfection of wildtype Dmp53 with a p53 luciferase reporter in Drosophila cells in culture resulted in transcriptional activation of the reporter that could be suppressed by adding increasing doses of the Sir2 activator resveratrol. Thus, dSir2 was shown directly to be a potent inhibitor of Dmp53 activity.

The experiments described above have facilitated the generation of a mechanistic model for how calorie restriction activates downstream pathways important in *Drosophila* longevity extension. The Helfand laboratory has postulated that calorie restriction acts in part through activation of dSir2 that in turn suppresses Dmp53 in the insulin-producing cells of the brain [20]. Dmp53 suppression results in reduced production of at least one critical insulin-like peptide (dILP2) by the insulin-producing cells. The reduced dILP production may regulate a number of downstream targets in the insulin signaling pathway, particularly in the fat body. Some of these targets, such as dFoxO, 4E-BP, and TOR have been shown to play roles in lifespan control in model organisms.

The key contribution of the series of *Drosophila* p53 papers from the Helfand laboratory is the placement of p53 as a key mediator of downstream CR signaling effects, in part through its effects on insulin signaling pathways. If confirmed by further studies, it will solidify a role for p53 as a longevity regulating gene. As always, exciting results such as these raise a number of new questions that beg to be addressed. For example, how does Dmp53 regulate dILP2 expression to effect downstream insulin signaling pathway effects? Are the DN Dmp53 effects solely through Dmp53 or are there other interaction targets? Which of the downstream insulin signaling pathway targets mediate the lifespan extension effects of activated dSir2 and suppressed Dmp53? The authors allude to CR effects that are dSir2 and Dmp53 independent. What are these and are they also insulin signaling independent? Does this model have applicability to mammalian systems? In mice, where the effects of Sir2 homologues on longevity are less clear and where adult tissue stem cells are believed to be an important component of aging, different pathways or cell types may be associated with CR-induced longevity effects. Unraveling these pathways in various model systems should provide profound new insights into the genetics and biology of aging and longevity.
